# Gene and Cell Therapy in Regenerative Medicine

**DOI:** 10.3390/cells15030212

**Published:** 2026-01-23

**Authors:** Albert A. Rizvanov, Ayşegül Doğan

**Affiliations:** 1 Institute of Fundamental Medicine and Biology, Kazan (Volga Region) Federal University, 420008 Kazan, Russia; 2 Division of Medical and Biological Sciences, Tatarstan Academy of Sciences, 420111 Kazan, Russia; 3 Genetics and Bioengineering Department, Faculty of Engineering and Natural Sciences, Yeditepe University, 34755 İstanbul, Türkiye

## 1. Introduction

Gene and cell therapies have become core components of regenerative medicine, moving from proof-of-concept studies toward clinically actionable strategies for repairing or replacing damaged tissues. In parallel with the expansion of approved advanced therapy medicinal products, the field is also redefining what “regeneration” means in practice—ranging from enhanced endogenous repair and functional recovery to cell replacement and, increasingly, engineered biological substitutes.

This Special Issue, “Gene and Cell Therapy in Regenerative Medicine” (https://www.mdpi.com/journal/cells/special_issues/GeneCellTherapy_Regenerative accessed on 16 January 2026), brings together 11 open-access publications (7 original research articles and 4 reviews) that reflect key directions in the field: (i) improvement in gene- and cell-product manufacturing workflows; (ii) understanding how tissue microenvironments shape therapeutic performance; (iii) integration of biomaterials with living therapeutics; and (iv) progress in delivery technologies and cell sources relevant to translational regenerative medicine.

## 2. Field Advances and Trends in 2024–2025

The 2024–2025 period has been marked by several developments that are directly relevant to regenerative medicine, particularly at the interface of gene delivery, cell replacement, immune engineering, and scalable manufacturing.

### 2.1. Expansion of Pluripotent Stem Cell-Derived Therapeutics and Movement Toward Later-Stage Development

A central trend has been the continued growth of human pluripotent stem cell (hPSC)-derived products in interventional trials. A 2025 landscape analysis reported 115 regulatory-approved clinical trials testing 83 hPSC products (as of December 2024), with >1200 patients dosed and no “generalizable” safety concerns identified to date—while emphasizing that long-term follow-up and product-specific risks remain essential considerations [1].

Type 1 diabetes (T1D) has emerged as a particularly informative indication for regenerative medicine because it requires durable engraftment, physiological responsiveness, and acceptable risk–benefit profiles. In 2024, device-based delivery of stem cell-derived β-cell precursors demonstrated that, in a subset of recipients, measurable C-peptide production could correlate with improved glucose-control metrics, while also highlighting the persistent challenges of cell survival, vascularization, and effective functional mass in humans [2].

In 2025, a multicenter study of an allogeneic stem cell-derived, fully differentiated islet-cell therapy (zimislecel) reported engraftment and functional C-peptide detection across treated participants, with clinically meaningful outcomes in the analyzed cohort, including insulin independence in a majority at one year (interim analyses), while also underscoring immunosuppression-related risks [3].

### 2.2. Immune Engineering as an Enabling Layer for “Off-the-Shelf” Regeneration

A major barrier for allogeneic cell replacement remains immune rejection. A key research direction in 2024–2025 has been immune engineering (hypoimmune or immune-evasive designs) intended to reduce or eliminate the need for chronic immunosuppression. In a 2024 primate-focused study, hypoimmune iPSC-derived products showed prolonged survival in immunocompetent, allogeneic settings, supporting the concept that immune engineering can function as a platform technology for scalable cell replacement [4].

### 2.3. Delivery Innovation and the Rise of In Vivo Editing as a Regenerative Tool

The regenerative impact of gene therapy is increasingly constrained (or enabled) by delivery. Beyond classic viral-vector paradigms, 2024 saw progress in lipid nanoparticle (LNP) reformulation strategies intended to achieve organ-targeted mRNA accumulation and translation while mitigating off-target distribution, which remains a critical translational bottleneck for systemic nucleic-acid therapeutics [5].

Concurrently, clinical data continue to strengthen the feasibility of in vivo genome editing with clinically relevant endpoints. In late 2024, a first-in-human study in ATTR cardiomyopathy reported rapid and durable reductions in circulating transthyretin following a single administration of a CRISPR-Cas9 therapy, illustrating the maturation of in vivo editing platforms that may ultimately be adapted to regenerative indications where durable pathway modulation is needed [6].

### 2.4. Regulatory Milestones Relevant to Tissue Repair Indications

Regulatory activity can reshape translational priorities. In June 2024, the FDA expanded approval of an AAV-based gene therapy for Duchenne muscular dystrophy (Elevidys) to include a broader population (ambulatory and non-ambulatory individuals aged ≥ 4 years), reflecting both the clinical demand for systemic gene delivery and the continuing evolution of evidence frameworks for high-need indications [7].

### 2.5. Xenotransplantation Enabled by Multiplex Gene Editing: Toward Organ Replacement Pathways

A distinct but closely related track of regenerative medicine is the development of gene-edited donor organs as a pragmatic response to organ shortages. In 2024, the first transplantation of a gene-edited pig kidney into a living human recipient was reported as a notable milestone for the field [8]. In 2025, FDA clearance for initial multi-patient clinical trials of genetically modified pig kidneys was reported, signaling movement from individual compassionate-use experiences toward structured clinical evaluation [9].

## 3. Overview of Contributions in This Special Issue

The published papers in this Special Issue can be grouped into three overlapping themes: (i) enabling technologies for gene and cell therapy manufacturing and deployment; (ii) cell therapy and microenvironmental determinants of regeneration; and (iii) reviews addressing delivery platforms and cell sources for translational regenerative medicine.

### 3.1. Enabling Strategies for Gene Therapy and Gene Delivery in Regenerative Contexts

Ex vivo expansion as a manufacturing lever for gene therapy: Fleischauer et al. investigated the use of the TGF-β inhibitor A83-01 to enhance murine hematopoietic stem and progenitor cell (HSPC) expansion, a relevant enabling step for gene therapy workflows where cell yield and functional preservation influence feasibility and cost. Such approaches are particularly important as the field seeks to standardize potency, improve transduction/editing consistency, and reduce variability in cell starting material [10].

BMP2 gene delivery for bone regeneration: Bukharova et al. compared adenovirus-based BMP2 gene delivery delivered in vivo versus ex vivo for bone regeneration. By explicitly contrasting these two routes, the study addresses a practical translational question: whether local in situ gene transfer or cell-mediated ex vivo delivery offers superior control over efficacy and safety in tissue repair settings [11].

Context-dependent responses in therapeutic angiogenesis/repair: Stafeev et al. evaluated combined HGF/VEGF gene therapy for limb ischemia in mice with impaired glucose tolerance, reporting a shift in regenerative response patterns. This work highlights how metabolic comorbidity can modify the balance between angiogenic, neurotrophic, and metabolic remodeling outcomes—an issue that remains central to real-world regenerative medicine, where patient heterogeneity frequently drives variable responses [12].

### 3.2. Cell Therapies, Tissue Microenvironments, and Biomaterial Support

Regeneration in sensory tissue via local cell administration: Ishikura et al. examined nasal administration of murine adipose-derived stem cells in a mouse model of olfactory epithelium damage. The study contributes to the broader theme that route of administration and local tissue context can be decisive for cell-therapy efficacy in neural and sensory regeneration [13].

Microenvironmental determinants in osteoarthritis-relevant cell therapy: Kitajima et al. showed that synovial fluid from human knee osteoarthritis can increase the viability of human adipose-derived stem cells, with mechanistic association to FOSL1 upregulation. These findings align with the growing view that “cell therapy performance” is not solely a property of the cell product but also of the disease microenvironment, supporting the rationale for patient stratification, preconditioning strategies, and microenvironment-aware potency assays [14].

Large-animal stromal cell biology and heterotopic tissue formation: Petinati et al. reported that porcine bone marrow multipotent mesenchymal stromal cells, implanted under the kidney capsule, can form an ectopic focus containing bone, hematopoietic stromal microenvironment, and muscle tissue. Such observations are relevant for translational model development, for understanding stromal cell plasticity in vivo, and for anticipating ectopic differentiation risks during regenerative applications [15].

Biomaterial–cell combinations for challenging wound-healing settings: Nie et al. investigated supramolecular hydrogel-wrapped gingival mesenchymal stem cells in cutaneous radiation injury. The work fits a broader trend toward “cell therapy plus matrix” designs, where biomaterials can improve local retention, viability, and paracrine persistence, while also enabling more controllable delivery in compromised tissues [16].

### 3.3. Reviews: Cell Sources, Omics-Informed Differentiation, and Gene Delivery Platforms

Red blood cells from hPSCs: Lee et al. reviewed progress in generating red blood cells from human pluripotent stem cells, emphasizing persistent challenges that are also relevant to other cell replacement products (maturation, scalability, and quality controls) [17].

Transcriptomics for differentiation control and quality assurance: Ogi and Jin discussed transcriptome-powered differentiation, reflecting the increasing role of single-cell and bulk transcriptomic frameworks for defining cell identity, detecting off-target states, and building quality-by-design pipelines for regenerative products [18].

AAV serotypes and gene therapy applications: Issa et al. summarized AAV serotypes and their use in gene therapy, a foundational topic for regenerative medicine where long-term expression and tissue tropism remain central design constraints [19].

Cell sources for retinal regeneration and translation challenges: Grigoryan reviewed cell sources for retinal regeneration and discussed translation-relevant considerations. Retinal indications remain one of the most active areas for regenerative development because they offer relatively accessible target tissues and quantifiable functional endpoints, while still posing major challenges in integration, durability, and immune compatibility [20].

## 4. Conclusions and Outlook

Across its research articles and reviews, this Special Issue underscores several recurring messages. First, regenerative outcomes depend on more than the selected vector or cell type: manufacturing constraints, delivery route, and tissue microenvironment can be equally decisive. Second, the field is moving toward platform thinking, where immune engineering, omics-driven quality control, and rational delivery design serve as reusable layers across multiple indications. Third, the most visible breakthroughs of 2024–2025—late-stage pluripotent stem cell therapeutics, improved delivery methods for nucleic acids, in vivo editing clinical data, and gene-edited organ replacement initiatives—collectively point to a near-term future in which regenerative medicine will increasingly be implemented as an engineered, combination technology rather than as a single modality.
